# Measurement of efficiency and its drivers in the Chilean banking industry

**DOI:** 10.1371/journal.pone.0300019

**Published:** 2024-05-20

**Authors:** Adriana Cobas, Alexandros Maziotis, Andres Villegas

**Affiliations:** 1 Department of Macrofinancial Modeling, Financial Policy Division, Central Bank of Chile, Santiago de Chile, Chile; 2 Departamento de Ingeniería Hidráulica y Ambiental, Pontificia Universidad Católica de Chile, Santiago de Chile, Chile; 3 Department of Business, New York College, Athens, Greece; 4 Escuela de Agronomía, Facultad de Recursos Naturales y Medicina Veterinaria, Universidad Santo Tomás, Santiago de Chile, Chile; Abu Dhabi University, UNITED ARAB EMIRATES

## Abstract

This paper estimates efficiency measures for the banking system in Chile for the period 2000-2019. In contrast to previous studies, we use input-distance functions, introduce the nonparametric slack-based model, and choose the intermediate inputs approach in determining inputs and outputs. Our results suggest that the Chilean system has achieved relatively high levels of efficiency, although with no significant variation over the sample period. Ownership (government, foreign and public) and size had a positive impact on efficiency. On average, mergers and acquisitions seem to have targeted highly efficient banks in order to improve the overall efficiency of the controlling institution in the short run. Other sources of efficiency gains could be an increase in bond funding or a reduction in expenses and capital holdings. The latter could be induced by deepening the local derivatives market.

## Introduction

The concept of efficiency refers to the best use of limited resources with minimum cost and maximum output. For the banking industry, an efficient performance allocates loans and financial assets with the minimum amount of deposits, capital and operative expenses (assuming a pure intermediation production function). The promotion of greater efficiency within the banking system is of interest to regulators, insofar as multiple studies conclude that it significantly contributes to virtuous processes such as the consolidation of the industry (in [1–3]), financial stability (in [4–8]), and even economic growth (as in [9–14]). Measuring the efficiency of a set of productive units, however, is a challenging objective due to its non-observable nature, the availability of multiple robust non converging methodological approaches, and the lack of consensus across the theoretical literature towards any of them.

Over the past thirty years, the Chilean banking system experienced several radical changes and yet sustained profitability levels that rank among the highest across countries with similar financial development levels (according to [15]). In the first place, a substantial consolidation process, reduced over 40 banks in the mid-1980s to 18 financial institutions in 2019. Simultaneously, an increase of international competitors occurred, there was an opening to public capitalization, regulatory innovations were set and new business models more reliant on technology emerged. The industry currently presents high profitability levels in international comparison, although with substantial heterogeneity, which might be explained by different levels of efficiency across banks.

In this paper we explore the determinants of efficiency in the Chilean banking system during 2000–2019. Our efficiency estimations employ parametric, semi and non-parametric techniques as a way to overcome the measurement controversy. In all cases we assume the intermediates approach, according to which deposits are an input instead of an output in the production function. We evaluate environmental drivers of efficiency gains such as merger and acquisition processes, both the public and state ownership of the institutions, the size of the units, and the origin of investing capitals. We also perform efficiency estimations at input level which allows to identify internal factors that explain institutional performance. Finally, we test whether efficiency can account for both the level and observed heterogeneity of system profits and, in addition, whether efficiency gains are directed toward increases in market share, dividend payments, or both.

### Literature review

This paper contributes to the literature analyzing efficiency, its determinants and implications in local banking systems in Latin America. Banking markets in the region have undergone significant changes since 1990 due to the gradual liberalization of capital markets, the internationalization of the banking industry, and the implementation of the Washington Consensus policies.

Comparative studies for the region show that the reforms mentioned above have helped to raise the average efficiency of local banking systems, particularly in terms of cost efficiency. However, there is considerable heterogeneity across countries. In particular, Chile, along with Colombia and Mexico, continues to operate at efficiency levels above those of the region [16]. In the same direction, [17] rejects overall convergence in the region and instead identifies two “convergence clubs” whose membership depends on the efficiency measure employed. There are also local studies for Brazil [18, 19], Ecuador [20–22], Peru [23], Argentina [24, 25], amongst others.

Several papers have attempted to measure bank efficiency in the Chilean banking sector. In general, they agree in characterizing the system as highly efficient. [26], based on the estimation of a cost function, argued that both economies of scale and economies of scope existed during the 1990s. However, efficiency gains due to economies of scale or scope were moderate and relevant only for the smallest banks. [27] found that the efficiency measures were robust to the use of parametric and nonparametric estimation techniques (Ordinary Least Squares, Seemingly Unrelated Regression, Stochastic Frontier Analysis, and Data Envelopment Analysis, respectively). In addition, they argue that all the different measures yielded increasing average efficiency levels that were above 75% in 1989–2001. [28] found that there were no significant differences from using alternative functional forms for the underlying technology in SFA. [29], based on a profit frontier analysis, emphasized that efficiency levels increased dramatically during 1987–2007, although their profit frontier measure was below that of other authors. Finally, [30], using a nonparametric technique, showed that the efficiency of the Chilean banking sector averaged around 85% in 2010–2014, with a U-shaped trajectory with a minimum in 2012.

In addition to the assessment with updated data, we make several contributions to the literature on bank efficiency in Chile. First, we use an input-distance function approach because it does not require price data, allows for multiple inputs and outputs simultaneously, and avoids biased estimates of input or output elasticities due to insufficient variation. This model also allows us to assess the impact of different operational characteristics, such as ownership type and mergers, on banks’ costs and efficiency. Second, we use a nonparametric slack-based model that measures efficiency at the input level using simultaneous contractions in inputs and expansions in outputs. Third, we use the intermediate inputs approach, which we believe is more consistent with current bank production technology as funding has become less physical capital intensive. Finally, unlike most studies (except [27]), we estimate and compare between parametric and nonparametric models to assess whether the choice of methodology is relevant to the empirical conclusions.

The remainder of the paper is organized as follows. The Section Materials and methods contains a first Subsection Discussion of efficiency measures where we present the main empirical alternatives to assess efficiency and discuss advantages and disadvantages of using each of them. Secondly, Subsection Efficiency models thoroughly presents each of the estimation models we used in the study. Section Results introduces the data used and presents empirical results. In Section Conclusion we include final considerations and conclusions.

## Materials and methods

### Discussion of efficiency measures

All empirical measures of efficiency coincide in the overall procedure: they combine outputs and inputs to build an efficient frontier for the industry across time and then score each productive unit according to the euclidean distance between that unit and the estimated frontier. The differences among techniques arise from the amount of structure imposed, and the assumptions made for both the frontier’s shape, and the error term’s distributional properties. The available estimation methods can be grouped into parametric and non-parametric techniques. In the specific case of bank efficiency, [31] survey 15,192 studies in 1998–2017. They find that 49% used non-parametric methods, 40% used parametric methods and the rest divide between semi-parametric methods and a mixture of techniques. In this section we briefly discuss the main features of each and briefly discuss their main features.

The Stochastic Frontier Analysis (SFA) (proposed by [32, 33]) is the most frequently used parametric technique. It consists of the econometric estimation of either a cost, production, or profit relationship among inputs and outputs. [34] summarize all these alternatives in Eq (1).

$$\begin{array}{c} y_{it}=x_{it}+\epsilon_{it} \end{array}$$ (1)

where *i* represents the productive unit, *y* stands for industry outputs (or costs or benefits), *x* is a vector of inputs. The error term is a composite in the form *ϵ*_*it*_ = *ε*_*it*_ + *u*_*it*_. The equation assumes a functional form to combine inputs, in general, a Cobb Douglas or a translog form. *u*_*it*_ represents total inefficiency and requires a distribution assumption (half normal, gamma, or truncated normal, uniform, beta and doubly truncated normal are the most used [31]). while *ε*_*it*_ captures random noise following the standard normal distribution. Conditioned by the noise term, any departure from the efficient level derives from the inefficiency score.

The main advantage of this method lies in the error term. First, the noise component allows for occasional deviations in efficiency due to unexpected events. Second, it admits the use of statistical inference on results.

The disadvantages originate in the assumptions required to run the model both the functional form of the interest relation and the distribution of the error term. For the underlying technology, translog, and Cobb-Douglas are the most widely used functional forms (other alternatives are Fourier flexible, composite or quadratic). The latter assumes constant returns to scale, can be used with a small number of observations and is easy to converge. The translog functional form assumes that the size of the companies varies and, then introducing multiple returns to scale, takes into account interaction among variables which allow the measurement of substitutability/complementarity among inputs and outputs and is easy to estimate. For the functional form selected, the results only hold if all the sample units use the exact same technology to combine inputs and outputs. This assumption might be very strong when sample pools contain banks that diverge in scale, market niche coverage, clientele, country, regulation, etc.

The non-parametric approach takes into account the productive unit heterogeneity by not assuming any frontier structure and eliminating any efficiency error. One of the procedures is Data Envelopment Analysis (DEA), which is defined in terms of [35], as a linear programming model wherein the frontier is determined by maximizing outputs, given available inputs (or minimizing inputs) and given total outputs. The frontier results from the best convex combination of factors according to one of these goals. It does not require the assumption of an efficiency error and its main advantage is simplicity at implementation.

The main drawback here is that the linear optimization technique is deterministic and does not include random errors. In terms of efficiency, it implies that there is no room for randomness, and as a consequence, any deviation from the frontier is completely attributed to efficiency gains or losses. Moreover, any measurement error or unintended productivity shock in each unit will compute the system frontier affecting all units’ scores in the sample. Also, it is very sensitive to extreme values. A second problem is the inability to directly include other explanatory variables such as controls. In order to face this restriction, [36] extended the bootstrap method on efficiency scores (more details in [37, 38]) and developed a double-bootstrap DEA (DBDEA) technique that allows statistical inference and biased-corrected scores efficiency.

The Slack Based Model (SBM) (proposed in [39]), is another non parametric technique. It shares with DEA the use of linear programming tools and the lack of an error term to allow for random deviations, thus inheriting all the advantages and disadvantages discussed above. An advantage over DEA is that it measures non radial efficiency (non linear) and identifies deviations of efficiency at input/output levels. The radial measure of efficiency in the previous models means that convergence towards the frontier implies a contraction of all inputs for a given level of outputs. In contrary, non radial models allow measuring an efficiency score for each input as well as potential savings.

In summary, all available methods to estimate efficiency present caveats. Parametric approaches can produce misspecification errors, which might drive over or underestimating efficiency. Non-parametric models attribute any deviation of the frontier to inefficiencies, avoiding economic, technical, or measurement shocks. The solutions proposed from the theoretical literature are to destructure parametric models or to randomize non-parametric ones (resampling). From an empirical point of view, although some authors signal that results from DEA or SFA are similar (cummins, casu), others recommend a parsimonious strategy to estimate using both methodologies first and then comparing them in robustness checks.

### Efficiency models

#### Stochastic frontier analysis

To estimate the stochastic frontier, we follow an input minimization approach in which banks minimize their inputs to obtain a determinate amount of output. Let’s assume that the production technology consists of a set of several inputs, *x*, that is used to produce a vector of outputs, *y*. This production technology can be represented by either the input distance function (developed by [40]). or the cost function approaches. In this case, we choose the first, which is a dual to cost function that overcomes the limitation of requiring input prices (Both data-intensive and hard to obtain [41]). The input distance function in Eq (2) measures the maximal radial contraction of all inputs for a given level of outputs [42]:

$$\begin{array}{c} D_{I}\left(x,y\right)\equiv max\left\{\theta :(\frac{x}{\theta },y)\in T,\theta >0\right\}\to D_{I}\left(x,y\right)=\frac{1}{TE} \end{array}$$ (2)

where *θ* denotes the technical efficiency score of each bank, which means that each bank can reduce the use of its resources by 1/*θ* and still produce the same level of output [43].

A bank is technically efficient when *D*_*I*_(*x*, *y*) = 1. If the bank is technically inefficient it follows that *D*_*I*_(*x*, *y*) = (1/*TE*) ≥ 1, where TE is Farrell’s input measure and *lnD*_*I*_(*x*, *y*) ≥ 0 is the technical inefficiency of the bank Ferro. The input distance is non-increasing in outputs, non-decreasing in inputs and linear homogeneous in inputs. (For the properties of the distance function please see [44–46]).

Following several studies (see for instance [41, 43, 47–49]), we use a translog functional form to present the technology as flexibly as possible and control for the different sizes of the banks (i.e. allowing economies of scale to vary) in Eq (3):

$$\begin{array}{cc} lnD_{I}\left(x,y\right)\; & =a_{j}+\sum_{l=1}^{L}a_{l}\;lny_{ljt}+\sum_{k=1}^{K}\beta_{k}\;lnx_{kjt}+\frac{1}{2}\sum_{l=1}^{L}\sum_{m=1}^{L}a_{lm}\;lny_{ljt}\;lny_{mjt}\\ \; & +\frac{1}{2}\sum_{k=1}^{K}\sum_{n=1}^{K}\beta_{kn}\;lnx_{kjt}\;lnx_{njt}+\sum_{l=1}^{L}\sum_{k=1}^{K}\gamma_{lk}\;lny_{ljt}\;lnx_{kjt}+\psi_{1}t\\ \; & +\frac{1}{2}\psi_{2}\;t^{2}+\sum_{k=1}^{K}\delta_{k}\;lnx_{kjt}t+\sum_{l=1}^{L}\xi_{l}\;lny_{ljt}\;t+\sum_{z=1}^{Z}\mu_{z}z_{jt}+\epsilon_{jt} \end{array}$$ (3)

where *j* represents the bank and *t* is the time period, *L* and *K* capture the total number of outputs and inputs, respectively, and *α*, *β*, *γ*, *μ*, *δ*, *ξ* are parameters that need to be estimated. The error is represented by *ϵ*_*jt*_ which follows the normal distribution, $\epsilon_{jt}∼N\left(0,\sigma_{\epsilon }^{2}\right)$. *a*_*j*_ are firm-specific effects and capture firm unobserved heterogeneity such as managerial inability. This unobserved heterogeneity is assumed to be fixed and time invariant [50]. This is the so-called “true” fixed effect model developed by [50] which separates unobserved heterogeneity from time varying technical inefficiency (please also see Eq (4)). Moreover, in expression (3) we include a set of control variables that could impact input requirements [51, 52].

After imposing homogeneity of degree 1 in inputs we get the final estimable form of the input distance function in Eq (4):

$$\begin{array}{ccc} -lnx_{Kj} & \; & =a_{i}+\sum_{l=1}^{L}a_{l}\;lny_{ljt}+\sum_{k=1}^{K-1}\beta_{k}\;lnx_{kjt}^{*}+\frac{1}{2}\sum_{l=1}^{L}\sum_{m=1}^{L}a_{lm}\;lny_{ljt}\;lny_{mjt}\\ \; & \; & +\frac{1}{2}\sum_{k=1}^{K-1}\sum_{n=1}^{K-1}\beta_{kn}\;lnx_{kjt}^{*}\;lnx_{njt}^{*}+\sum_{l=1}^{L}\sum_{k=1}^{K-1}\gamma_{lk}\;lny_{ljt}\;lnx_{kjt}^{*}\;\psi_{1}t\\ \; & \; & +\frac{1}{2}\psi_{2}t^{2}+\sum_{k=1}^{K=1}\delta_{k}\;lnx_{kjt}^{*}t+\sum_{l=1}^{L}\xi_{l}\;lny_{ljt}t+\sum_{z=1}^{Z}\mu_{z}\;z_{jt}+\epsilon_{jt}-u_{jt} \end{array}$$ (4)

where $x_{kj}^{*}=x_{kj}/x_{Kj}$ and *u*_*jt*_ is the technical inefficiency of each bank *j* at any time *t* and is assumed to follow the exponential distribution, $u_{jt}∼\text{Exp}\left(\sigma_{u}^{2}\right)$. Technical efficiency of each bank *j* at any time *t* is calculated as $TE_{jt}=e^{u_{jt}}$.

Thus, Eq (4) is the final SFA model because it includes both noise and inefficiency. This model is estimated using maximum likelihood estimation (MLE) techniques ([47, 50]). Finally, the restrictions needed for the imposition of the homogeneity in +1 in inputs are as follows [43]:

$$\begin{array}{c} \sum_{k=1}^{K}\beta_{k}=1,\;\sum_{n=1}^{K}\beta_{kn}=0\;\forall k,\sum_{l=1}^{L}\gamma_{lk}=0\;\forall l,\sum_{k=1}^{K}\delta_{k}=0\;\forall k \end{array}$$ (5)

Finally, we impose conditions in (6) for symmetry [41]:

$$\begin{array}{c} a_{lm}=a_{ml}\;\forall m,\;\beta_{kn}=\beta_{nk}\;\forall n \end{array}$$ (6)

#### DEA

Following [53–57], an input-oriented DEA model was adopted because bankers focus more on inputs than outputs. Since the banking sector deals with government regulations and imperfect competition causing deviation from an optimal scale [58], variable returns to scale (VRS) is considered to be more appropriate in relation to scale assumption.

An input-oriented VRS DEA model is employed to estimate efficiency scores as follows Banker 1984:

$$\begin{array}{c} \begin{array}{c} Min\;\theta_{j}\\ s.t.\\ \sum_{k=1}^{N}\gamma_{k}x_{ik}\leq \theta_{j}x_{ij},\;1\leq i\leq S\\ \sum_{k=1}^{N}\gamma_{k}y_{rk}\geq y_{rj},\;1\leq r\leq L\\ \sum_{k=1}^{N}\gamma_{k}=1\\ \gamma_{k}\geq 0,\;1\leq k\leq N \end{array} \end{array}$$ (7)

where *θ*_*j*_ represents whether banks are technically efficient (*θ*_*j*_ = 1) or not (*θ*_*j*_ < 1), (1−*θ*_*j*_) indicates the proportional reduction of inputs that a bank can achieve given a certain level of output, *N* is the number of banks and *γ* is a *n* × 1 vector of constant.

#### Double boostrap DEA

To analyze how environmental variables may explain variations in bank efficiency, we apply a double bootstrap approach. This technique was proposed by [36] and consists in a truncated pooled regression with a bootstrap to avoid the dependency problem (serial correlation between the error term and the environmental variables) and to obtain valid estimates in the second stage of the regression analysis. The model has the following econometric specification:

$$\begin{array}{c} {\hat{\theta }}_{j}=z_{j}\beta +D^{t}\gamma +\epsilon_{j},\;\;j=1,...,N \end{array}$$

where the bank’s efficiency scores (${\hat{\theta }}_{j}$) calculated in Eq (7) are regressed against several environmental variables *z*_*j*_ considering time $D^{t}\;\left(t=1,...N\right)$ as a vector of dummies. Finally we assume that the distribution of $\epsilon_{j}∼N\left(0,\sigma_{\mu }^{2}\right)$, is truncated normal with zero mean, unknown variance, and left truncation at 1 − *x*_*j*_*β*. Considering the information on the parametric structure and distributional assumption we construct the truncated maximum likelihood function and maximize it with respect to $\left(\beta ,\gamma ,\sigma_{\mu }^{2}\right)$, given our data.

#### Slacks based model

We define two vectors *v*^+^ ≥ 0 and *v*^−^ ≥ 0, called slacks, that indicate input excess or output shortfall for each productive unit with inputs *x*_0_ and outputs *y*_0_:

$$\begin{array}{c} \begin{array}{c} x_{0}=\sum_{i}x_{i}\gamma_{i}+v^{-}\\ y_{0}=\sum_{i}y_{i}\gamma_{i}+v^{+} \end{array} \end{array}$$ (8)

An SBM is used to calculate the efficiency and the slack value. The following index *κ*

$$\begin{array}{c} \kappa =\frac{1-\frac{1}{S}\sum_{i=1}^{S}v_{i}^{-}/x_{i0}}{1+\frac{1}{L}\sum_{r=1}^{L}v_{i}^{+}/y_{i0}} \end{array}$$ (9)

is defined in terms of the amount of slack, and has value between 0 and 1.

Finally, efficiency measure obtains from minimizing Eq (9) subject to conditions in Eq (8) and considering λ ≥ 0, *v*^+^ ≥ 0, *v*^−^ ≥ 0.

The formula in (9) can be transformed into (10), where the ratios in the numerator and denominator indicate inputs and outputs inefficiencies respectively:

$$\begin{array}{c} \kappa =\frac{\frac{1}{S}\sum_{i=1}^{S}\left(x_{io}-v_{i}^{-}\right)/x_{io}}{\frac{1}{L}\sum_{r=1}^{L}\left(y_{ro}+v_{r}^{+}\right)/y_{ro}} \end{array}$$ (10)

According to [39, 59], we can modify the denominator of the measure *κ* by introducing a small positive number, *φ* = 10^−6^, as:

$$\begin{array}{c} \kappa =\frac{1-\frac{1}{S}\sum_{i=1}^{S}v_{i}^{-}/x_{i0}}{1+\frac{\phi }{L}\sum_{r=1}^{L}v_{i}^{+}/y_{i0}} \end{array}$$ (11)

This modification corresponds to the input-oriented model which establishes more relevance on the input slacks than the output slacks.

### Data

We construct a data panel using the monthly balance sheet and income statements of twenty Chilean banks from 2000 to 2019, available at the Comisión para el Mercado Financiero (CMF). Each series has 240 observations, except in cases where corporate events occurred. For more details on bank data, see the section 1 in S1 Appendix.

All income statement variables are 12 months accumulated, and the whole set of variables was deflated using July 2005 = 100, which minimizes the distance to period’s average inflation. The sample was truncated at 2019 to avoid possible interferences in regular system dynamics that might have introduced government liquidity injection programs during COVID-19 pandemic. (See [60] for a detailed analysis of Chilean alternative credit policies during 2020 and 2021).

We focus on the group of 20 commercial banks clustered as big, medium and consumption in [61] (see section 1 in S1 Appendix for the detail of institutions by category). Although the authors identify four other clusters (treasuries, foreign trade, financial instruments, medium specialized), we disregarded them because they are smaller and more specialized institutions, less connected to the economy as a whole.

Inputs and outputs, were selected applying the intermediates approach (first proposed in [63]) most frequent in banking efficiency literature. This approach assumes that banks produce financial intermediation, and as a consequence, inputs in their production function are the funding they receive from different sources and outputs are the allocations of the funds in multiple investment alternatives, such as credits or market assets. The alternative production approach assumes both deposits and credits are outputs of the production function while employees and fixed assets are the principal inputs (this method has mostly been used to compare attained efficiency between branches within an institution).

Table 1 lists the variables used across the models. We categorized as inputs deposits, regulatory capital, issued bonds and operating expenses (the total of salaries, buildings rent, and utilities plus interests paid). The outputs are loans and other earning assets (liquid assets plus financial investments). Finally, we have other variables such as cluster, ownership in several dimensions (public, state, foreign), inflation, GDP growth (using the monthly IMACEC Index at the Central Bank of Chile). For the cases of merger and acquisitions, we have a dummy which takes the value one during the year of occurrence of the event.

**Table 1:** Sample description.

**Continuous Variables**					
**Variable**	**Units**	**Observations**	**Avge.**	**SD**	**In models**
Operating expenses	CLP mm	3683	287	299	Inputs
Deposits	CLP mm	3683	3210	3780	Inputs
Capital	CLP mm	3683	574	694	Inputs
Issued Bonds	CLP mm	3683	677	1220	Inputs
Loans	CLP mm	3683	4330	5190	Outputs
Financial earning assets	CLP mm	3683	1500	1880	Outputs
IMACEC	Index	3683	84	18	Controls

**Categorical Variables**			
**Variable**	**Category**	**Observations**	**% of Obs.**
Cluster	Big	1015	28
	Medium	2006	54
	Consumption	662	18
State owned	Yes	240	6.5
	No	3443	93.5
Foreign ownership	Yes	1331	36
	No	2352	64
Public Traded	Yes	1652	45
	No	2031	55

Average exchange rate 2000 − 2019 is CLP1.00 = USD586.47.

## Results

### Stochastic frontier analysis

Table 2.a presents the estimated results from the input distance function. Variables were normalized around their mean so that the first-order estimated coefficients of inputs and outputs can be interpreted as elasticities at the sample mean. As expected, output elasticities have a negative sign and are statistically significant, which means that increases in outputs cut the distance to the frontier [63]. Input elasticities have a positive sign and are statistically significant from zero, so that increases in inputs augment the distance to the frontier. From these results, we can then conclude that the input distance function is both non-increasing in outputs and non-decreasing in inputs which implies that the model is well-specified [63].

**Table 2:** Empirical model results.

2.a SFA model, dependent variable:			2.b DBDEA, dependent variable:		
Opex			DEA scores		
Frontier	Coefficient	St.Er.	Frontier	Coefficient	St.Er.
Loans	-0.8712***	0.0096	Medium	-0.2698	0.0124
Fin. ear. as.	-0.1366***	0.0064	Consumption	-0.4679	0.0139
Deposits	0.4251***	0.0147	State	0.1115***	0.0244
Capital	0.3991***	0.0142	Foreign	0.0825***	0.0075
Bonds	0.0284***	0.0032	Public Trading	0.0002	0.0062
Loans 2	-0.1509***	0.0059	M&A ocurred	0.1301	0.1139
Fin. ear. as. 2	-0.0558***	0.0037	L3.M&A ocurred	0.2007*	0.1088
Loans*Fin. ear. as.	0.0677***	0.0043	L6.M&A ocurred	0.0983	0.0926
Deposits 2	0.0320***	0.0009	L12.M&A ocurred	0.0493	0.0695
Capital 2	-0.0063	0.0154	2001.year	-0.0185	0.0194
Bonds 2	0.0028***	0.0003	2002.year	-0.0138	0.0210
Deposits*Loans	0.0316***	0.0040	2003.year	0.0187	0.0238
Deposits*Fin. ear. as.	0.0018	0.0051	2004.year	0.0847***	0.0322
Capital*Loans	0.0158**	0.0075	2005.year	0.0511	0.0389
Capital*Fin. ear. as.	0.0059	0.0063	2006.year	0.0080	0.0496
Bonds*Loans	0.0058***	0.0004	2007.year	0.0068	0.0572
Bonds*Fin. ear. as.	-0.0024***	0.0003	2008.year	-0.1131*	0.0645
Deposits*Capital	-0.0829***	0.0089	2009.year	-0.0901	0.0619
Deposits*Bonds	-0.0053***	0.0007	2010.year	0.0378	0.0721
Capital*Bonds	0.0023***	0.0007	2011.year	-0.0548	0.0854
Loans*Time	0.0007	0.0006	2012.year	-0.0955	0.0972
Fin. ear. as.*Time	-0.0009*	0.0006	2013.year	-0.0840	0.1063
Deposits*Time	0.0060***	0.0012	2014.year	-0.1227	0.1100
Capital*Time	-0.0037***	0.0012	2015.year	-0.1221	0.1152
Bonds*Time	-0.0002***	0.0000	2016.year	-0.1046	0.1190
Time	-0.0003	0.0005	2017.year	-0.0691	0.1229
Time 2	-0.0001	0.0003	2018.year	-0.0498	0.1336
Medium	-0.0589**	0.0250	2019.year	-0.0857	0.1362
Consumption	-0.2234***	0.0528	cons	1.0175***	0.1398
State	0.1071***	0.0250	*σ*	0.1320***	0.0025
5–7 Foreign	0.1436***	0.0123			
Public trading	0.0969***	0.0143			
Imacec	0.0004	0.0003			
M&A ocurred	0.0693**	0.0293			
L3.M&A ocurred	0.0394	0.0269			
L6.M&A ocurred	0.0126	0.0260			
L12.M&A ocurred	0.0066	0.0234			

*** Coefficients are statistically significant from zero at the 1% level.

** Coefficients are statistically significant from zero at the 5% level.

* Coefficients are statistically significant from zero at the 10%

From the elasticities of loans and financial earning assets we find that, keeping other things equal, a 1% increase in loans and financial earning assets could lead to average increases in operating costs of 0.8712% and 0.1366%, respectively. This finding suggests that loans are a major cost driver for the banking sector.

The elasticities of deposits, capital, bonds, and operating expenditures suggest that deposits and capital are major determinants of input requirements in the Chilean banking sector.

Both loans and financial earning assets have been increasing costs at a rate of 15% and 5.6%, respectively as indicated by the negative sign of their squared term. The positive sign in the interaction term between loans and financial earning assets, which is significant, signals a cost complementarity between them. On average, an increase in the provision of more loans could lead to an increase in financial earning assets, which in turn could lead to lower costs and thus push up the performance of the bank. Moreover, on average, as deposits grow its elasticity increases; whereas, the opposite is true for capital as indicated by the squared terms of these variables.

Increases in loans are accompanied by increases in deposits and capital as indicated by the statistically significant and positive sign of their cross input-output terms (not like the the case of financial earning assets, which result is non significant). Consequently, this could lead to lower input requirements on average which could have a positive impact on the performance of the bank. In other words, banks could increase their efficiency by reducing the capital holdings on their balance sheets. This capital in excess of their legal requirements has been signaled in several occasions by the central bank (see for example [64]). A plausible explanation is that facing an underdeveloped and illiquid local derivatives market banks hedge credit and market risk using capital that then accumulates in excess on their balance sheets. As a result, there is some scope for efficiency gains through capital reduction. (The which would also. A similar result is observed from the interaction term between bonds and loans.

Furthermore, the interaction term between both deposits and capital, and deposits and bonds is negative and statistically significant from zero. This means that there were some substitution possibilities between these two inputs that might not have led to lower costs on average.

The time variable that captures technical change was not statistically significant and has a negative sign, which means that the banking sector experienced a technical regress of 0.03% per year on average. The same occurs in the case of technical regress with a positive, although non-significant, coefficient of 0.01%. Moreover, the interaction term of deposits with time is positive; whereas, the interaction term between capital and bonds with time is negative meaning that technical change has been deposits saving and capital and bonds using.

Finally, we evaluate the robustness of the efficiency measure to different model specifications and data sets. The results are presented in the section 2 of S1 Appendix. The alternative technical inefficiency assumptions (*u*_*jt*_ in Eq (4)) tested include truncated (model 2) and half-normal (model 3) distributions, presented in Table A2 of S1 Appendix. Overall, the sign and significance of the coefficients remain consistent across the different specifications. Only the interactions between deposits and financial assets and between capital and loans deviate from previous results. Thus, the model is consistent with the assumption of technical inefficiency.

Table A3 of S1 Appendix shows the response of our results to different data sets. For this purpose, we have introduced variables successively according to their nature: production variables (model 4), interactions between production variables (model 5), and finally time components (model 6 and model 7) and environmental variables (model 8). The conclusions drawn earlier appear to be quite robust after the addition of non-linear (interaction and quadratic terms) and time components. This highlights the importance of including non-linearities and time in models of technological progress.

The discussion on the control variables, cluster, ownership and merger and acquisition, is located in subsection Efficiency drivers using a multimodel approach along with DBDEA results on these matters.

### Efficiency drivers using a multimodel approach

In this section we use our estimations to analyze the evolution of efficiency at both aggregate and disaggregate levels, the effect of environmental variables (such as size and ownership) and of corporate events. In all cases we use both parametric and non parametric estimates in order to identify eventual implications of methodology in results.

#### System efficiency

**Fig 1:**
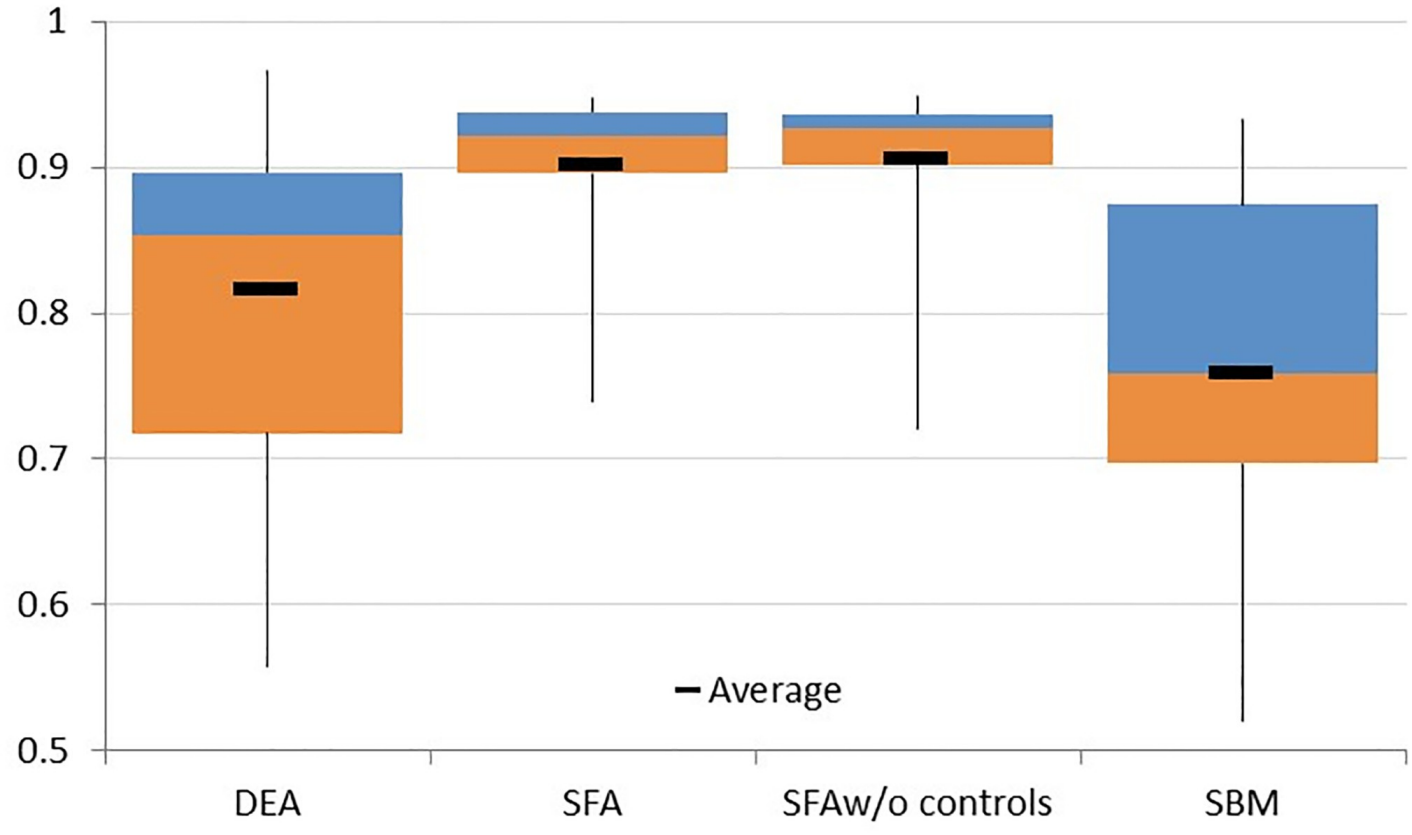
Boxplots of overall efficiency 2000–2019 across models. Boxes represent interquartilic range. Colors change at median value.

The Chilean banking system performs in the top quarter efficiency range across DEA, SFA, SFA without controls and SBM models as depicted in Fig 1, leaving some room for improvement. Average system efficiency ranges between 75% in SBM and 90% in the SFA model. We also estimated the SFA model without other explanatory variables for a fair comparison with non parametric models. Nevertheless, this estimate yielded only negligible differences with the general version, as the figure shows. An interesting feature is that in both models mean and median diverge in the last decade, which will discuss below.

In addition, as in [35] parametric models present more concentrated results than non parametric models. In effect, the SFA with and without controls achieves an interquartile range between 3.5% and 4.2% while in parametric models it situated above 17%. Moreover, dispersion originates in the lower portion of the distribution. While maximums across models gather between 93.3% and 96.6% minimums spread between 51% and 73%, the lowest in the SBM and DEA models.

**Fig 2:**
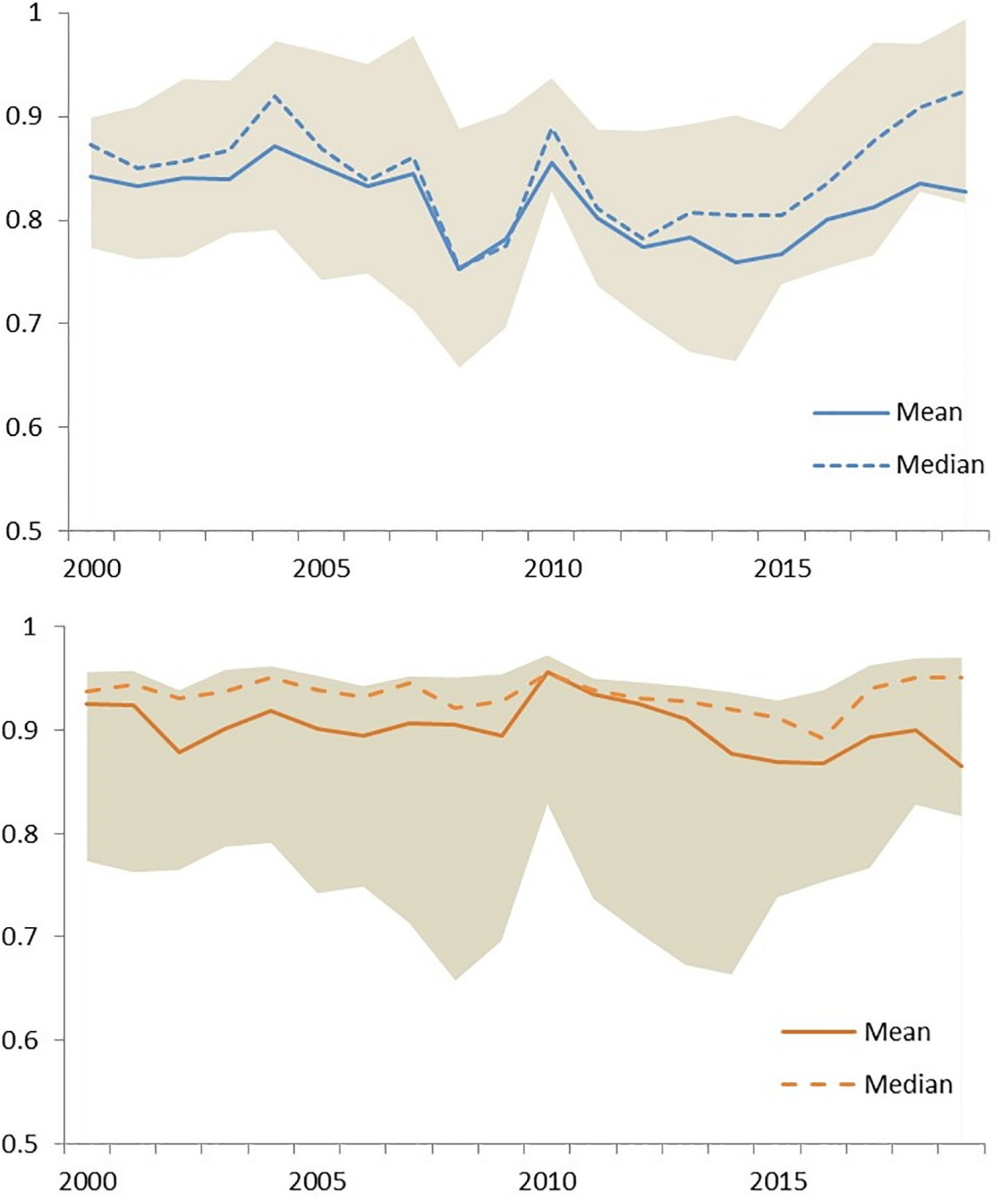
System efficiency dynamics 2000–2019. a) DEA model results. b) SFA model results.

There is no evident time trend when system performance is analyzed through the whole period as depicted in Fig 2. Only in the case of the DEA estimation in panel Fig 2.a the median efficiency exhibits a consistent increase after 2012. On the contrary, mean efficiency reduces with persistence after 2010 in the SFA model in Fig 2.b. Estimations in both models suggest there is an important impact in year 2010 coincident with an important regulatory reform in the Chilean banking system.

#### Efficiency at institutional level

Efficiency models allow us to construct efficiency scores and rankings. As observed above, strong similarities exist between non parametric results and significant differences between them and the parametric results. In Table 3 we present a set of cross correlations of both efficiency scores and institutional rankings using annual averages. For instance, the correlation between the DEA and SBM scores and rankings is 90% and 92% respectively (Table 3.a and 3.b respectively). On the contrary, the DEA and SFA correlation reduces to 42% when scores are considered and reduce further to 22% when rankings are observed instead (Table 3.a and 3.b respectively).

**Table 3:** Correlations between efficiency scores and efficiency rankings.

3.a Efficiency vectors				3.b Ranking vectors			
	**DEA**	**SFA**	**SBM**		**DEA**	**SFA**	**SBM**
**DEA**	1	-	-	**DEA**	1	-	-
**SFA**	0.42	1	-	**SFA**	0.22	1	-
**SBM**	0.90	0.39	1	**SBM**	0.92	0.26	1
3.c Top 5 ranking				3.d Bottom 5 ranking			
	**DEA**	**SFA**	**SBM**		**DEA**	**SFA**	**SBM**
**DEA**	1	-	-	**DEA**	1	-	-
**SFA**	-0.60	1	-	**SFA**	0.51	1	-
**SBM**	0.41	0.19	1	**SBM**	0.43	-0.3	1

In addition, we evaluated the correlations in efficiency rankings in the top and bottom five institutions across models. In this case we used average efficiency along the 2000–2019 period. As depicted in Table 3.c and 3.d, DEA and BSM present the highest correlation in the top five institutions of the ranking and a high correlation in the bottom five institutions (around 40% in both cases). The DEA and SFA are negatively correlated in the top list and highly positively correlated in the bottom one (-60% and 51% respectively). Finally the BSM and SFA models show the lowest correlations at both top and bottom rankings (19% and 25% respectively).

**Fig 3:**
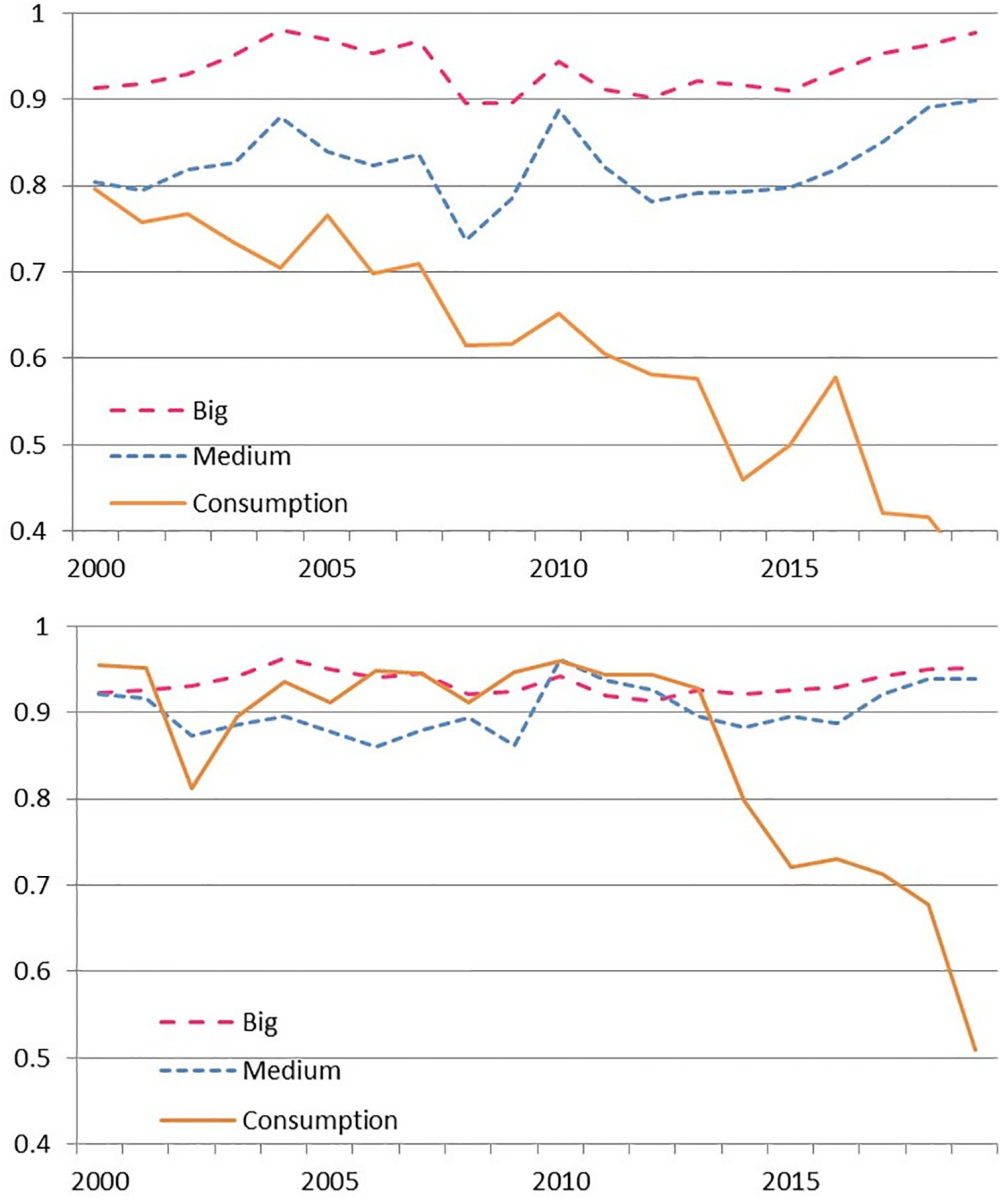
Average efficiency by cluster. a) DEA model. b) SFA model.

Focusing on institutional performance, we identify a significant heterogeneity by cluster hidden in the aggregate measure (as it evidences Fig 3). Despite the differences across models, big and medium banks achieve the highest efficiency levels and converge in the last decade. In these groups, efficiency differs 11% on average in DEA while the gap reduces to 3% in SFA, in both cases dominated by big banks during most of the period. Meanwhile consumption banks stand out for being the worst performers with a sharp drop in the most recent term. Increasing inefficiency persists along the whole period in DEA but only after 2010 in SFA. Note that efficiency obtained with SBM at cluster level, in section 3 of S1 Appendix, preserves the features observed in the other models. (In Subsection Efficiency disaggregated at input level, we discuss on the determinants of this phenomenon).

SFA results suggest that the system reached a high level of efficiency, with an average of 0.90 in the last two decades. Nevertheless, we observe a significant heterogeneity across units which that renders an average interquartile range of almost 0.03 in the sample. The heterogeneity produced divergent time trends when we consider average or median performances.

#### Environmental effects

We now discuss the effects of size and ownership using the SFA and BDEA models, presented in Table 2.a and 2.b. In both models, negative and significant coefficients in medium and consumption banks indicate that size impacts on system efficiency. In particular, efficiency reaches the highest rates in big banks, followed by medium and consumption institutions.

State ownership dummy is significant and positive (in line with results in sepu), which means that state banks are on average 11% more efficient than private banks in both estimated models. Foreign ownership also has a significant positive effect on efficiency, increasing it by 14% in the SFA model and 8.25% in DBDEA. This result aligns with findings by [65] who found that Chinese banks in 1994–2003 were more efficient if owned by foreigners, although there is plenty evidence of the opposite case. (See for instance [66, 67]).

Regarding publically traded shares, we found a positive effect in both models but not significant in the case of DBDEA. According to [35] firms with stockholder ownership face stronger incentives to control costs or increase efficiency, and if they do so we should expect them to be more efficient. Nevertheless, empirical results are mixed. (See [68] for a positive relationship in Turkish banking system).

#### Mergers and acquisitions

We found a positive impact of merger and acquisitions on overall efficiency although with mixed results in terms of timing. The SFA model suggests that mergers had a positive effect on efficiency during the year of occurrence (at 10% significance), but there are no significant impacts on the following three quarters. The DBDEA model, on the contrary, does not report impact at occurrence but only one quarter after it (at 10% significance) and again with a positive sign. That said, it is important to note that both models suggest that effects impact the controlling institution only in the short term.

Overall, the results suggest that on average, the merger and acquisition strategy targeted already efficient institutions benefiting directly the controller institution at purchase (a negative sign would have suggested that on average low efficient institutions are targeted by more efficient institutions). The same result was found for instance by [4] in US banking system during the eighties, where the probability of being acquired was lower for cost inefficient banks.

#### Summary of multi model results

We find that methodology impacts system average estimate locating parametric models results above those of no parametric ones. Nevertheless, models coincided in that none detected any clear time trend over the period. A direct comparison of correlations among the efficiency scores shows that non parametric measures are quite similar among themselves and different from parametric results. We then observed cluster averages, and found that DEA reports higher heterogeneity in efficiency levels and scores, but ordering varies in 2000–2012 when comparing against SFA.

Regarding environmental effects, both models obtain the same directions in all significant variables (only in the case of public trading did we obtain mixed results at significance). Regarding mergers and acquisitions, both models identify an impact but disagree on its timing. The SFA model detects a response just as an event occurs, while DBDEA reports effects in the following first quarter.

In summary, we found that model selection has an impact on the resulting level of efficiency, which is mainly a consequence of their being based on different assumptions. However, model estimations were indistinctly capable of identifying the relationship between efficiency and other variables in most of the econometric tests we performed.

Note that our results seem to avoid endogeneity bias due to the environmental variables (bank clusters, mergers and acquisitions, and state, foreign, and public ownership), as they are determined independently of the efficiency level. However, this is not the case for the production variables, i.e. although our inputs and outputs are common in the literature and most are independently determined (deposits and loans), some may have endogeneity problems. For example, if banks raise capital or issue bonds for a general efficiency improvement project. To address this issue, it may be appropriate to use instrumental variable models.

### Efficiency disaggregated at input level

**Fig 4:**
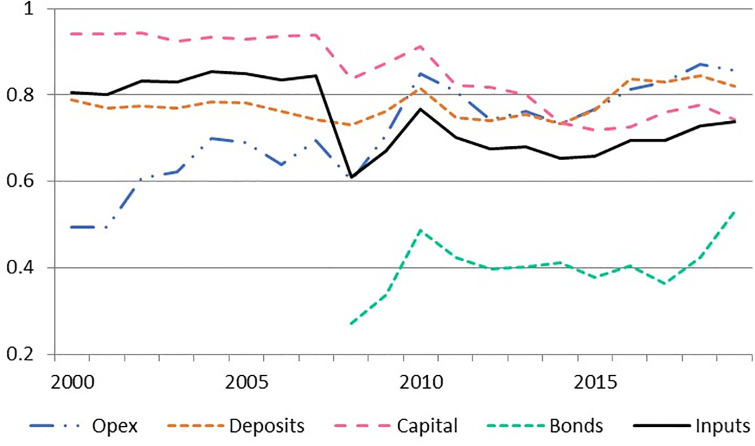
Input average efficiency SBM during 2000–2019.

The SBM results show a significant heterogeneity in the efficiency of the considered inputs, both in levels and trends as depicted in Table 4 and in Fig 4. Considering the system as a whole, the Chilean banking industry can improve its efficiency by reducing its inputs by 24% (1 − 0.76). In particular, the optimal combination of adjustments consists of reducing opex by 33% (1 − 0.67), deposits by 23% (1 − 0.77), capital by 13% (1 − 0.87) and bonds by 27% (1 − 0.73).

**Table 4:** Inputs efficiency resulting from SBM (averages).

Efficiency level	Op. Exp.	Deposits	Capital	Bonds	Inputs
System	0.67	0.77	0.87	0.73	0.76
Big	0.75	0.96	0.95	0.86	0.88
Medium	0.74	0.80	0.82	0.64	0.75
Consumption	0.36	0.43	0.86	0.77	0.60

At cluster level, Big and Medium banks achieve the best performance in deposits and capital leaving room for improvement in operative expenses and bonds (especially in the case of Medium banks). The group of Consumption banks underperforms in all inputs, in particular in operative expenses and deposits, being the last one the main efficiency driver inside the inputs set (see Subsection).

The most notable trend is in operative expenses which present a steady increase in efficiency almost duplicating its level over the whole sample (Fig 4). The efficiency in the use of deposits is nearly stable across the period at 80%, while capital efficiency, that starts at a very high level in 2000 (94%) follows a persistent decrease ending in 2019 at 74%. Note that the use of bonds as a “novel” source of funds remains at low efficiency levels (in the 30% to 50% range) which explains an overall efficiency decrease when data is pooled after 2008. Nonetheless bond efficiency presented a sharp augment after 2016.

In summary capital and bonds, in particular the latter, show room for efficiency improvement in the overall sample. Deposits remain highly efficient and operative expenses present the most significant increase in efficiency in the period. Let us notice what happens with each component at cluster level.

**Fig 5:**
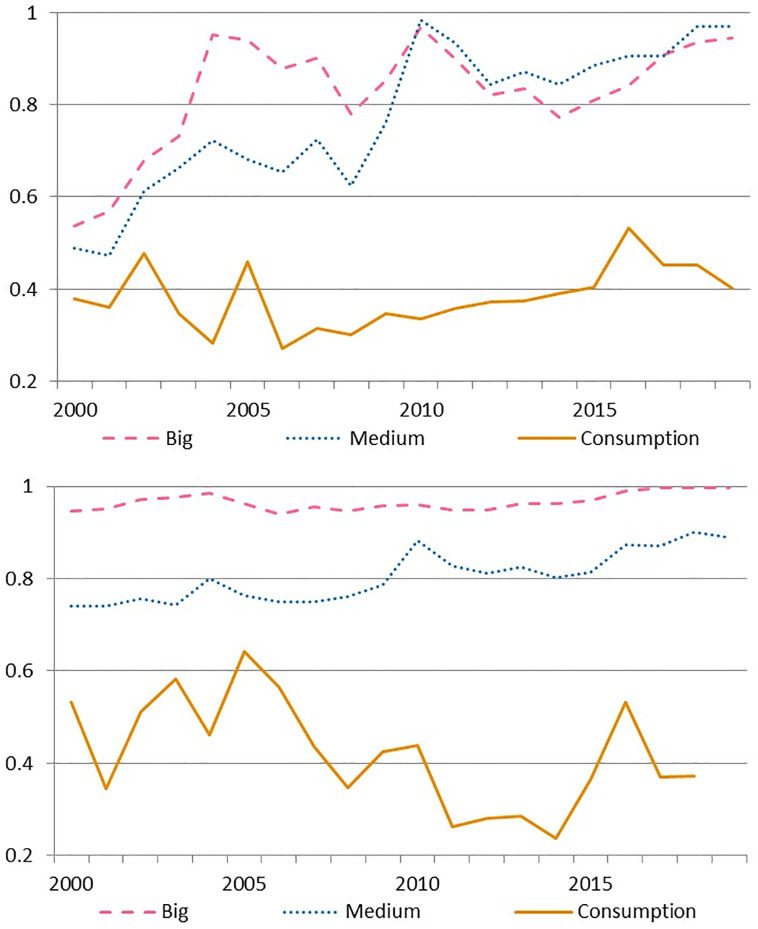
Cluster average efficiency in inputs with SBM during 2000–2019. a) Operative expenses. b) Deposits.

Operative expenses have been a major driver for efficiency growth in the case of Big and Medium banks across the whole period, according to Fig 5.a. Both groups present a sharp persistent increase and converge to very high levels at the end of the sample which contrasts with the dynamics at the Consumption banks that do not evidence a clear time pattern in the long term, and persist at low efficiency levels during the whole sample.

In the case of deposits, Fig 5.b depicts three very different patterns. Big banks sustain at very high levels, with a slight increase to end up the period at the frontier. Regarding the Medium banks, they achieve an 10% improvement over the sample yet situating below the previous ones. On the contrary, the performance of the Consumption cluster does not show a clear time pattern. Digging further, we find that Consumption banks experience the steepest upwards trend in their share of sight deposits, going from an average of 3.2% before 2010 to 13.8% after that year (a total six fold across the sample). For the Chilean banking industry, that means an increase in legal reserves and as a consequence less funding available per unit of deposit for both lending or investing. In particular, in the case of Chile, sight deposits require 9% of legal reserves, while term deposits only require 3.6%. (In section 4 of S1 Appendix we present a comparison for the trajectory of the share of sight deposits by cluster).

**Fig 6:**
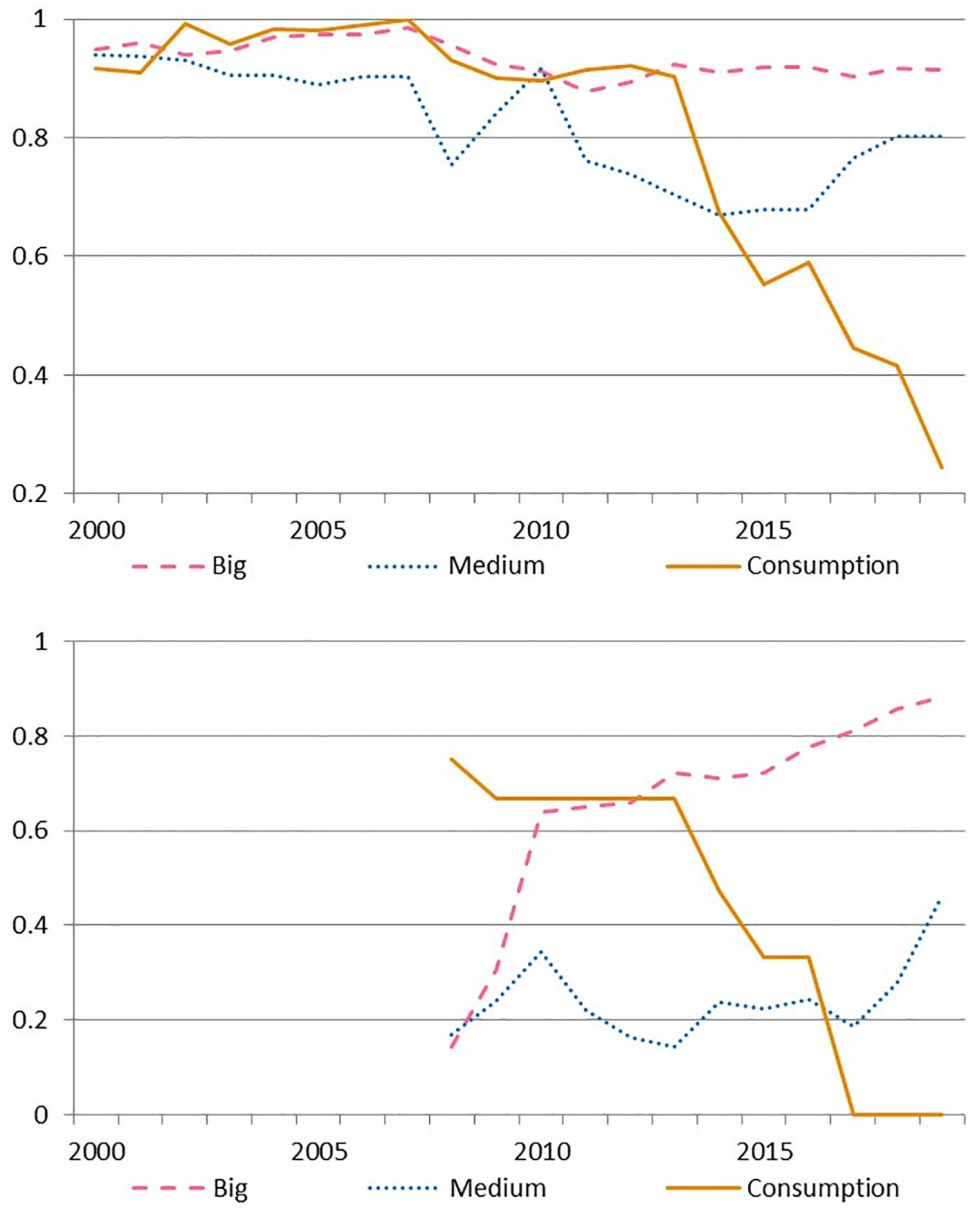
Cluster average efficiency in inputs with the SBM during 2000–2019. a) Capital. b) Bonds.

Capital efficiency, in Fig 6.a, is the first occasion in which all clusters present a decreasing trend. The effect is the mildest among Big banks, where efficiency reduces only 3% and yet remains above 90% at the end of the period. That is not the case for Medium banks in which capital efficiency reduces from 94% in 2000 to 80% in 2019. But Consumption banks, in the extreme, experience a capital efficiency plummeting of almost 80% across the sample accelerating the drop after 2013. Our hypothesis for this group of banks is that as it is strongly related to retail activity (all these banks are part of retail conglomerates) and thus to consumption credit, they obtain high returns even while keeping capital in excess (which might be necessary in case of a negative shock increasing non performing loans in riskier credit portfolio). In fact, the return on equity of the cluster persistently overperformed that of the other groups since 2015 while keeping high capital levels. The riskier activity, which requires more capital, reflects on higher rates of provisions to assets. Indeed Consumption banks doubled their system level of provisions to assets in 2005–2015 and started to converge towards them only after 2015. (See section 5 in S1 Appendix for more detail).

In the case of bonds efficiency, we have data only in the period 2007–2019, as shown in Fig 6.b. We found that efficiency has been increasing steadily in the case of Big banks, which coincidentally behave as the most active group in the bond market. Note that while the average ratio of issued bonds to assets in 2010–2019 reached 14% and 16% in Big and Medium banks respectively, it located in 8% in the case of Consumption banks (with a maximum level of 13.6% in September 2018 followed by a contraction to 9.5% afterwards). A recent increase in bonds efficiency exists in the case of Medium banks although it remains at half the efficiency level of the Big banks. Finally, for Consumption banks, our model again signals excess funding strategy, resulting in the low efficiency in bonds, which deepens throughout the available data.

In summary, at the inputs level, the cluster of Big banks sustained the highest efficiency across the period, with significant improvements from operative expenses and the issuance of bonds for extra funding. Medium banks remained 10% below due mostly to a lower efficiency on deposits, capital and loans (yet, in most cases converging toward the previously mentioned group). Consumption banks present low efficient levels in the dimensions this paper assumed, mostly as a consequence both of deposit behavior and excess capitalization strategy.

### Efficiency effect on market share and returns

In the previous subsections, we obtained measures of efficiency and examined its main determinants. In this last subsection, we will empirically assess how efficiency gains or losses affect other system variables, such as market share and dividend distribution policies.

#### Efficiency and return heterogeneity

In section 6 of S1 Appendix we present our results that monthly efficiency helps predict monthly return on equity (Granger precedence). Seeking to learn whether this precedence extends to the heterogeneity dimension we want to know whether efficiency dispersion explains return dispersion. We estimated and compared standard deviations of return on equity and efficiency scores. Table 5 presents testing results in both cases using year and bank fixed effects.

**Table 5:** Empirical model results.

5.a SFA model, dependent variable: standard deviation(roe)			5.b DBDEA, dependent variable: standard deviation(roe)		
Variable	Coefficient	St.Er.	Variable	Coefficient	St.Er.
sd(Efficiency)	14.5698***	2.4761	sd(Efficiency)	-77.1124***	7.4489
FE bank	yes	-	FE bank	yes	-
FE year	yes	-	FE year	yes	-

Using SFA efficiency we obtain a positive significant relationship, which means that the more banks differentiate themselves in efficiency, the more they differ in observed return on equity in the studied period. DEA efficiency, however, obtains a negative result. A possible explanation for this divergence derives from differences in estimation among both models. Indeed the SFA uses panel data for estimation while the BDEA pools monthly bank data.

#### Efficiency dividends and market share

We then asked whether efficiency improvements are applied to price reductions in order to gain market share, or, if in contrast, efficiency gains (losses) are fully channeled towards to shareholders’ dividends with little impact on the relation to customers. This second hypothesis would contribute to explain high return in the Chilean industry when compared with peer markets.

Thus, we tested Granger causality pairing efficiency with the variables dividend payments (and dividend policy) and market share using annual data (in section 6 of S1 Appendix we reproduce the test with monthly data substituting dividend policy with ROE). Note that this test does not inform about causality in the regular sense of the term but instead responds to how much each variable in the pair helps predict the other, and then we can say it precedes or “Granger causes” it.

We used the Non Granger Causality test in [69] suitable for unbalanced panel data. Results for up to 2 and 3 lags (maximum available according to sample) in each case are presented in Table 6. All variables are stationary according to panel data Fisher-type test based on Augmented Dickey Fuller. Note that the lag time limit restricts us to testing at most medium term relations, not long term plans.

**Table 6:** Panel granger non-causality test, $\tilde{Z}$ values. Annual data.

6.a Test using DEA efficiency measure						
**max x** **lags y**	**dividends Eff. DEA**	**Eff. DEA dividends**	**d.policy Eff. DEA**	**Eff. DEA d.policy**	**mktsh Eff. DEA**	**Eff. DEA mktsh**
1	30.2500***	-0.8964	2.6065***	1.9281*	2.3337***	0.3803
2	7.5250***	-0.1730	2.1330**	-0.5369	4.0402***	-0.5137
3	-	-	-	-	1.7982*	-1.0765
6.b Test using SFA efficiency measure						
**max x** **lags y**	**dividends Eff. SFA**	**Eff. SFA dividends**	**d.policy Eff. SFA**	**Eff. SFA d.policy**	**mktsh Eff. SFA**	**Eff. SFA mktsh**
1	12.9060***	-0.4051	0.9952	-0.6289	1.0303	0.4476
2	6.2504***	0.9696	-0.3816	-1.5383	1.0660	0.4147
3	-	-	-	-	0.3802	-0.6558

*H*_0_: *x* does not Granger cause *y*. *H*_1_: Granger Causality for at least one individual.

***: *p* < 0.01,

**: *p* < 0.05,

*: *p* < 0.1.

Regarding the relationship between efficiency and dividends, both models fail to identify a precedence relation from efficiency to dividends and only the DEA detects a weakly significant relation at one lag. This result suggests that dividend payments are complex decisions depending on multiple variables other than efficiency itself (such as capital accumulation decisions, economic expectations, policy restrictions, etc). As a consequence, efficiency gains or loses are insufficient to predict them, while they do predict returns (see section 6 in S1 Appendix).

However, Granger causation in the relation goes in the opposite direction, that is from dividends and dividend policy to efficiency. We find this to be an unexpected and striking result. A possible explanation, is that both variables might be Granger caused by a third one but at different time stages. For instance, would it be loans, an increase in loans determines an increase on return for higher interest revenues and possibly higher dividends. At the same time, higher loans increase our efficiency measure which then appears to be preceded by dividends.

Finally, the DEA efficiency score is Granger caused by market share (although his result may be related to our measure of market share as the percentage of market assets thus relating it to size).

In summary, with the DEA measure of efficiency and data up to three lags, we found that market share predicts efficiency, and efficiency predicts returns. This causal relationship aligns with the Relative Market Power hypothesis, which suggests that firms with substantial market shares strive to achieve higher levels of efficiency, allowing them to generate supernormal profits. [70] with data from 1995–2005, previously discarded this hypothesis, which suggests that now the system has transitioned from an early to a mature consolidation stage.

## Conclusion

In this paper we investigate the determinants of efficiency in the Chilean banking system during the period 2000–2019. To this end, we contribute to the the existing literature by using distance functions, the intermediates approach, the introduction of the slacks based model and the estimation and comparison between different estimation methods.

We found significant heterogeneity across measures, mostly between parametric and nonparametric techniques, which needs to be addressed in subsequent work. However, this did not have a significant impact on the empirical tests of environmental variables, trend, or M A outcomes.

The Chilean banking system achieves high levels of efficiency in all estimated models. According to our measures, large and medium banks are the best performers, with the latter converging to the former throughout the sample. This is not the case for the consumption cluster, which shows inefficient performance by most measures. A breakdown by input points to operating expenses as the main driver of efficiency gains in the sample, while capital shows a downward trend over the same period.

In addition, our results suggest that size as well as, foreign and state ownership have a positive significant impact on efficiency. Public trading, also positive, showed significant results only at the SFA specification. Mergers and acquisitions showed positive significant short term results. This suggests that expansions were directed towards already efficient institutions, in order to increase efficiency in the whole new entity (among other objectives). The heterogeneity of efficiency has an impact on the observed heterogeneity on returns, although our models yielded opposed directions.

Finally, non causality tests were run between efficiency, market share, return and dividends to identify precedence relations between such variables. Our results depend on the efficiency series employed. In the case of DEA efficiency, we find that efficiency dynamics precede the return on equity up to seven lags while the impact on market share only occurs after one quarter. Short term causality from market share to efficiency does not surprises us, as assets are part of inputs. Regarding SFA efficiency we found bidirectional causality in all the tested pairs. Finally, we obtained some unexpected results when testing dividends and dividend policy. Future analysis should dig deeper to better understand the relationship among these variables.

As noted above, regulators may be interested in promoting bank efficiency for several reasons. One of them is the direct acquisition of already efficient institutions by the more lagging ones. This appears to be a more complicated option as systemic consolidation has reached a mature stage. Another, but only for retail banks, is to work on costs, as medium and large banks have done successfully. A third way to increase efficiency could be to deepen bond funding, the efficiency of which seems to lag behind other inputs. This source of funding is cheaper (in terms of network and advertising) than deposits, and can be raised more quickly than increasing the depositor base. However, it depends on the depth of the capital market and the alternative uses that these investments offer to investors in addition to the return on open positions (for example, liquidity and the possibility of using them as collateral in repo markets). Thus, regulators may be interested in considering feedback effects between the expansion of capital markets and the further sophistication and efficiency of the banking system for macroeconomic growth. Finally, midsize and consumer banks have plenty of room to improve capital efficiency. To the extent that capital slack is a tool for managing credit risk, bank managers could evaluate alternative risk hedging tools (such as derivatives) to free up capital for more efficient uses.

This work could be extended in several research directions. One is to incorporate interesting innovative methods, such as estimation with bad outputs (for example, for non-performing loans) or the application of the dual DEA approach [71], which resolves the ambiguity of the approaches (intermediate or production). Second, to study the impact of recent events on the efficiency of the system, such as the contingency policy for the COVID-19 pandemic or the expansion of cooperation and competence relationships with the fintech sector. Finally, it may be of interest to analyze the relationship between efficiency, profits and risk.

## Supporting information

S1 Fig Boxplots of overall efficiency 2000–2019 across models. Boxes represent interquartilic range. Colors change at median value.
(JPG)

S2 Fig System efficiency dynamics 2000–2019. a) DEA model results. b) SFA model results.
(JPG)

S3 Fig Average efficiency by cluster. a) DEA model. b) SFA model.
(JPG)

S4 Fig Input average efficiency SBM during 2000–2019. (JPG)

S5 Fig Cluster average efficiency in inputs with SBM during 2000–2019. a) Operative expenses. b) Deposits.
(JPG)

S6 Fig Cluster average efficiency in inputs with the SBM during 2000–2019. a) Capital. b) Bonds.
(JPG)

S7 Fig Cluster average efficiency with SBM during 2000–2019. (JPG)

S8 Fig Sight deposits as a share of total deposits, cluster average. (JPG)

S9 Fig Return on equity, cluster average. (JPG)

S10 Fig Ratio provisions to assets, cluster average. (JPG)

S1 Appendix (PDF)
