# Supplementary figures and images for: Measurement of efficiency and its drivers in the Chilean banking industry
Source: PLoS One. 2024 May 20;19(5):e0300019. doi: 10.1371/journal.pone.0300019 (PMC11104684; doi:10.1371/journal.pone.0300019)

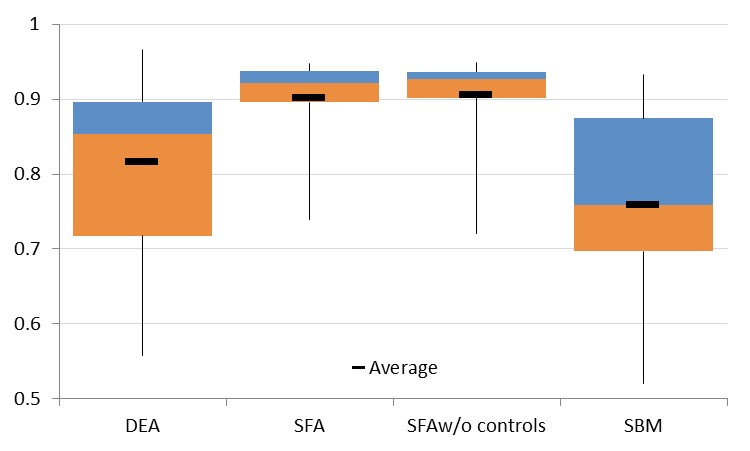

Supplement: S1 Fig — Boxes represent interquartilic range. Colors change at median value. (JPG) [file pone.0300019.s001.jpg]

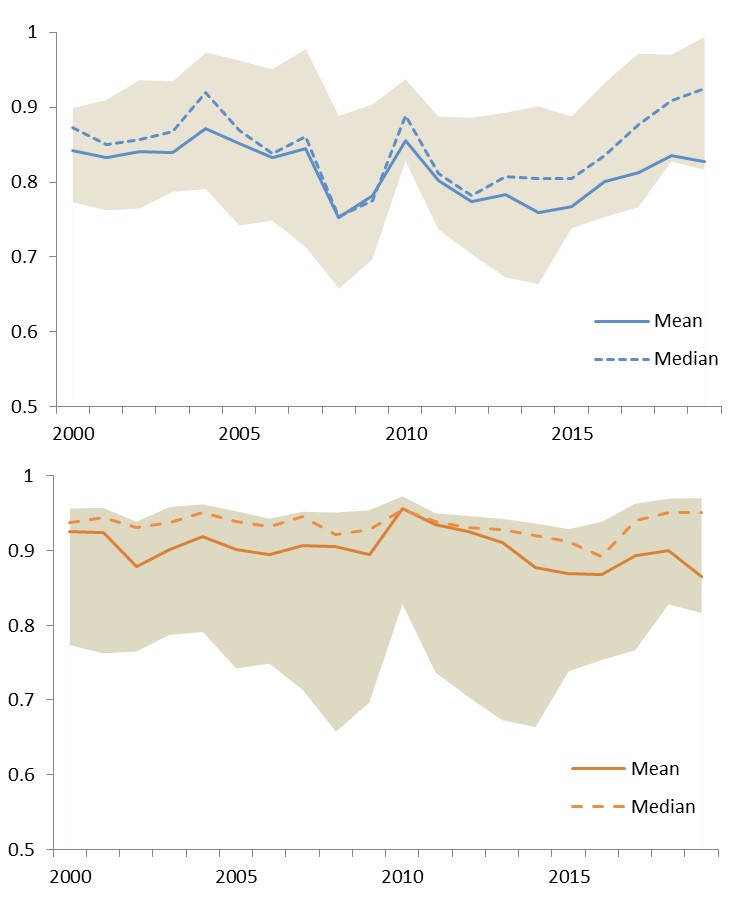

Supplement: S2 Fig — a) DEA model results. b) SFA model results. (JPG) [file pone.0300019.s002.jpg]

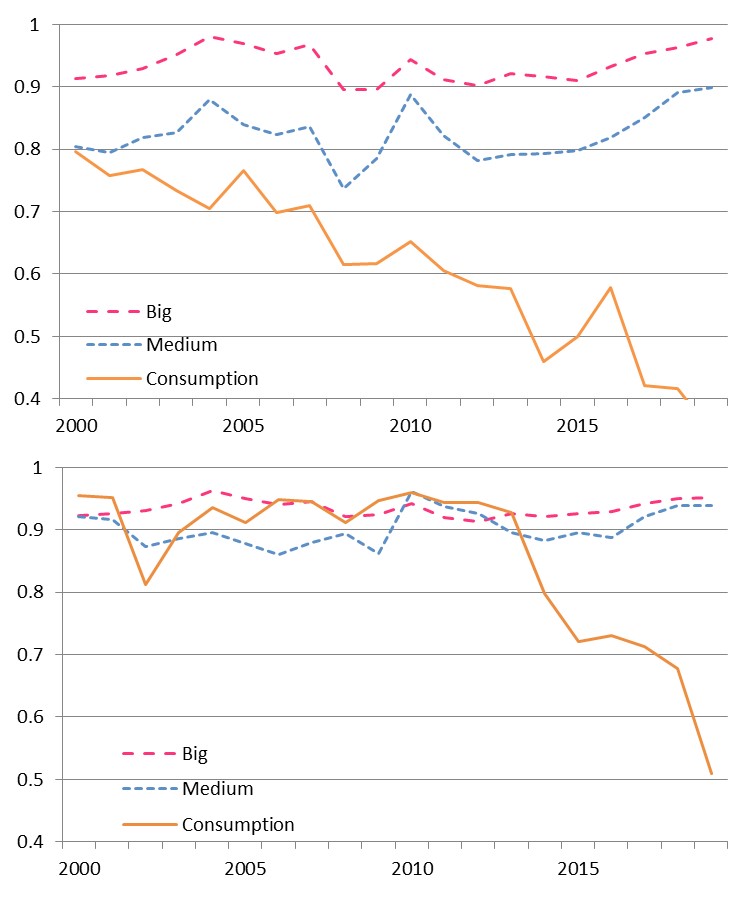

Supplement: S3 Fig — a) DEA model. b) SFA model. (JPG) [file pone.0300019.s003.jpg]

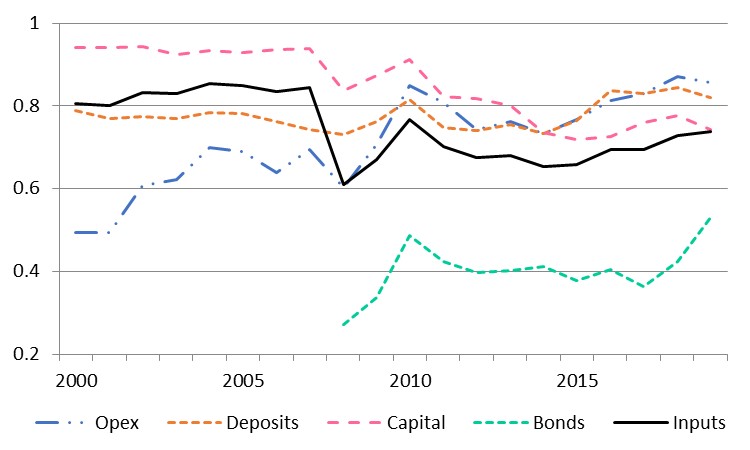

Supplement: S4 Fig — (JPG) [file pone.0300019.s004.jpg]

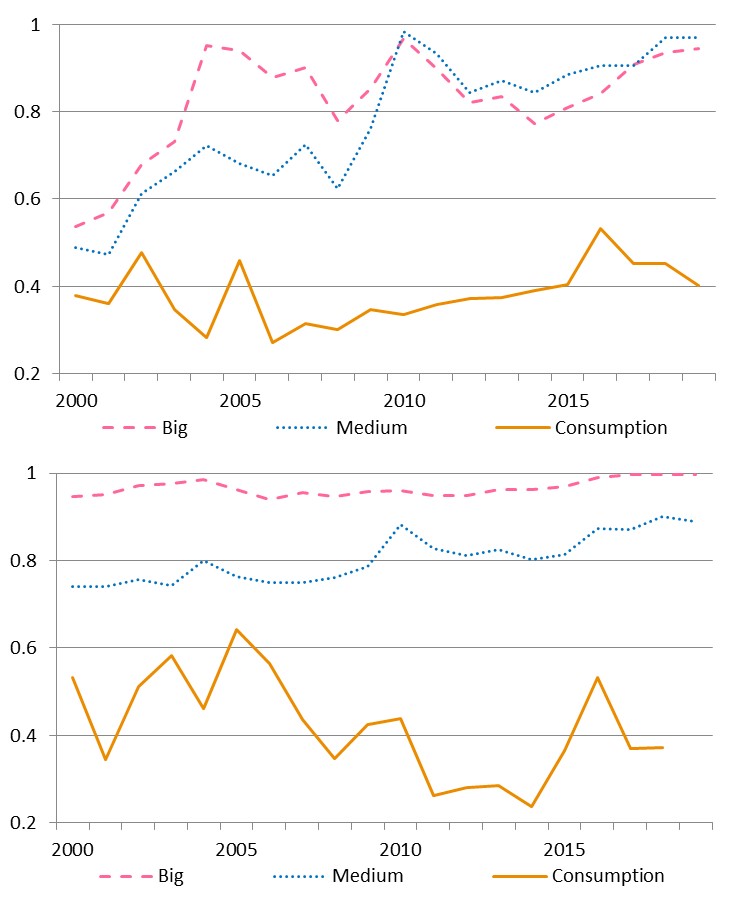

Supplement: S5 Fig — a) Operative expenses. b) Deposits. (JPG) [file pone.0300019.s005.jpg]

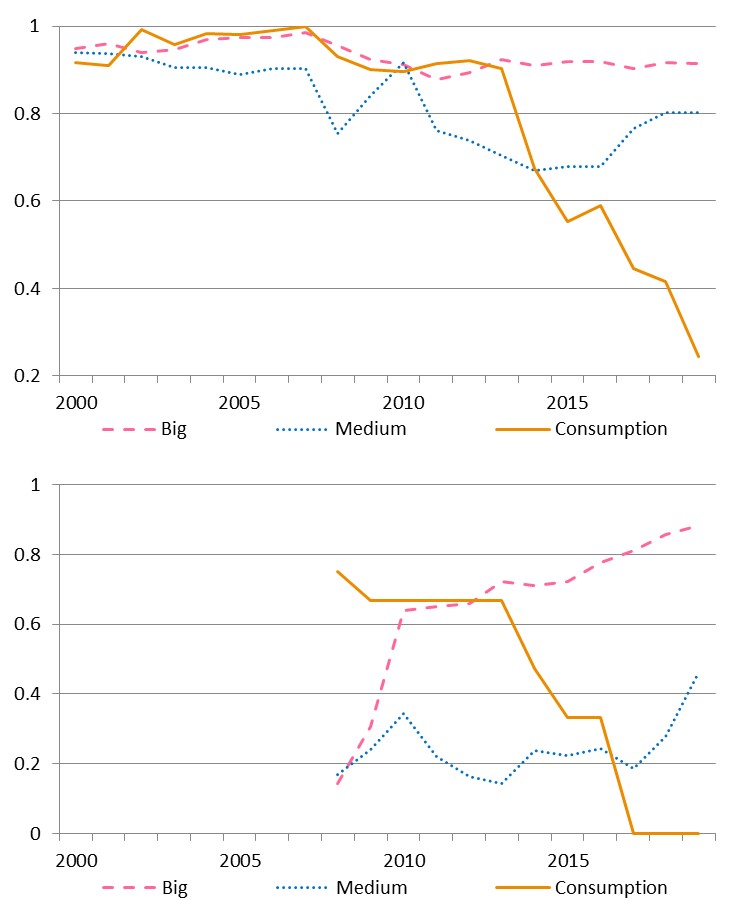

Supplement: S6 Fig — a) Capital. b) Bonds. (JPG) [file pone.0300019.s006.jpg]

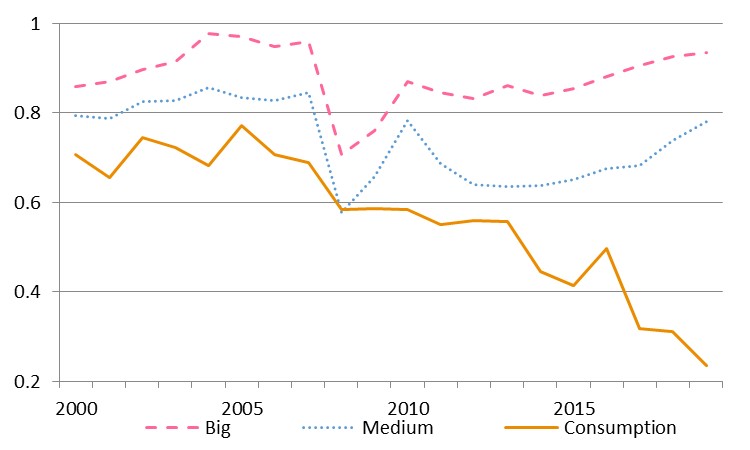

Supplement: S7 Fig — (JPG) [file pone.0300019.s007.jpg]

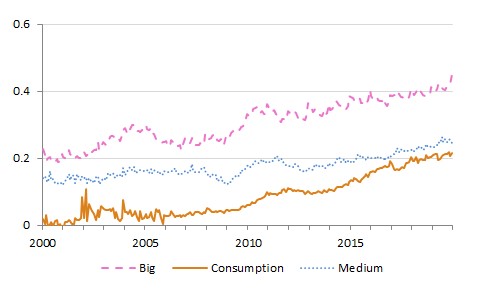

Supplement: S8 Fig — (JPG) [file pone.0300019.s008.jpg]

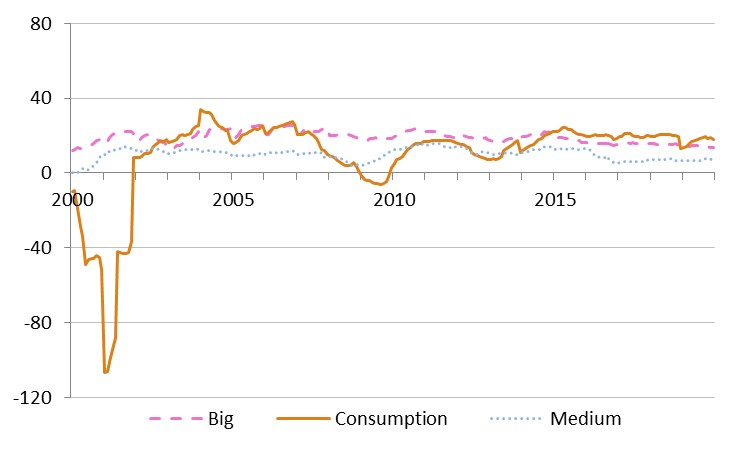

Supplement: S9 Fig — (JPG) [file pone.0300019.s009.jpg]

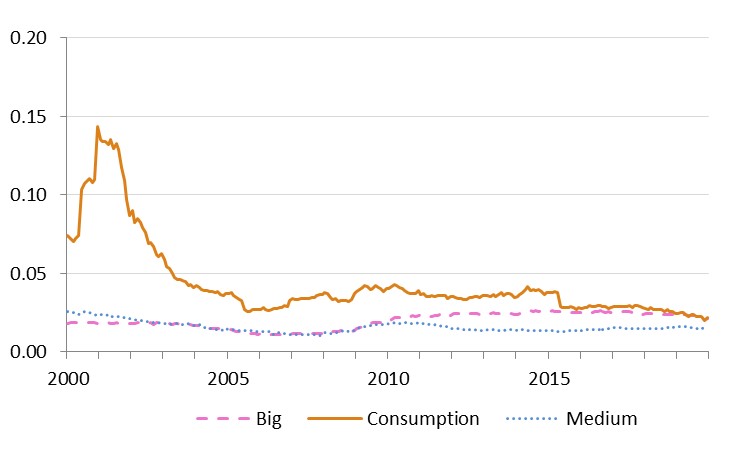

Supplement: S10 Fig — (JPG) [file pone.0300019.s010.jpg]
