# Supplementary material for: Measurement of efficiency and its drivers in the Chilean banking industry
Source: PLoS One. 2024 May 20;19(5):e0300019. doi: 10.1371/journal.pone.0300019 (PMC11104684; doi:10.1371/journal.pone.0300019)
Supplement: S1 Appendix — (PDF) [file pone.0300019.s011.pdf]

1 Classification of local banks in clusters

Table A1 presents the banks considered in the analysis, the cluster assigned in [?], and the available months of data. Note that in the case of the Consumption cluster, it is characterized by both its size (as the smallest banks) and its commercial strategy differentiation, as these banks specialize in the consumption segment by assembling credit over a strong retail commerce activity.

Table A1. Chilean banks according to industry clusters and total available months of data.

**Big banks**	**T**	**Medium banks**	**T**	**Consumption**	**T**
BCI	240	BBVA	224	Conosur	66
Edwards	24	BICE	240	Falabella	240
Chile	240	Citibank	96	Paris	144
Estado	240	Corpbanca	95	Ripley	212
Santander	240	Itau	240		
Santiago	31	Desarrollo	118		
		Scotiabank	240		
		Internacional	240		
		Rabobank	173		
		Security	240		

The series with less than 240 months correspond to institutions that experienced corporate events. The cases of fusions are: Citibank and Chile (2007), Edwards and Chile (2002), Santiago and Santander (2003). Cases of acquisitions: Scotiabank to Desarrollo (2010), Scotiabank to Paris (2016), Scoriabank to Rabobank (2012), Scotiabank to BBVA (2017).

2 Robustness checks on model requirements and data sets

Table A2. Model specifications under different technical inefficiency assumptions

**Frontier**	**Model 1**	**Model 2**	**Model 3**
Loans	-0.8712***	-0.8218***	-0.8338***
Fin. ear. as.	-0.1366***	-0.1398***	-0.1429***
Deposits	0.4251***	0.3804***	0.4450***
Capital	0.3991***	0.2596***	0.3762***
Bonds	0.0284***	0.0638***	0.0299***
Loans$\hat{2}$	-0.1509***	-0.1874***	-0.1505***
Fin. ear. as.$\hat{2}$	-0.0558***	-0.0651***	-0.0568***
Loans*Fin. ear. as.	0.0677***	0.0802***	0.0683***
Deposits$\hat{2}$	0.0320***	0.0314***	0.0318***
Capital$\hat{2}$	-0.0063	-0.1253***	-0.001
Bonds$\hat{2}$	0.0028***	0.0061***	0.0029***
Deposits*Loans	0.0316***	0.0536***	0.0343***
Deposits*Fin. ear. as.	0.0018	-0.0236***	0.0077
Capital*Loans	0.0158*	-0.0844***	0.0086
Capital*Fin. ear. as.	0.0059	0.0394***	0.0036
Bonds*Loans	0.0058***	0.0097***	0.0066***
Bonds*Fin. ear. as.	-0.0024***	-0.0020***	-0.0028***
Deposits*Capital	-0.0829***	-0.0057	-0.0872***
Deposits*Bonds	-0.0053***	-0.0065***	-0.0044***
Capital*Bonds	0.0023**	-0.0015	0.0013
Loans*Time	0.0007	0.0011	0.0006
Fin. ear. as.*Time	-0.0009	-0.0012	-0.0008
Deposits*Time	0.0060***	0.0035**	0.0053***
Capital*Time	-0.0037**	-0.0003	-0.0031*
Bonds*Time	-0.0002***	-0.0001*	-0.0002***
Time	-0.0003	-0.0001	-0.0002
Time$\hat{2}$	-0.0001	0.0002	-0.0002
Medium	-0.0589	-0.0409	-0.1215***
Consumption	-0.2234***	-0.2067**	-0.2826
State	0.1071***	0.1401	0.0199
Foreign	0.1436***	0.1881***	0.0662***
Public trading	0.0969***	0.1149***	0.0005
Imacec	0.0004	-0.0003	-0.0006
M&A	0.0693*	0.1096**	0.0611*
L3.M&A	0.0394	0.0742	0.0332
L6.M&A	0.0126	0.0292	0.0045
L12.M&A	0.0066	-0.0027	0.0003

*** Coefficients are statistically significant from zero at the 1% level. ** Coefficients are statistically significant from zero at the 5%. * Coefficients are statistically . significant from zero at the 10%.

Table A3. Model specifications under different dataset

**Frontier**	**Model 4**	**Model 5**	**Model 6**	**Model 7**	**Model 8**
Loans	-0.7745***	-0.8241***	-0.8297***	-0.8297***	-0.8712***
Fin. ear. as.	-0.0831***	-0.1685***	-0.1653***	-0.1651***	-0.1366***
Deposits	0.0201***	0.5286***	0.5307***	0.5306***	0.4251***
Capital	0.6953***	0.3074***	0.3092***	0.3096***	0.3991***
Bonds	-0.0061***	0.0385***	0.0378***	0.0378***	0.0284***
Loans$\hat{2}$		-0.1701***	-0.1718***	-0.1719***	-0.1509***
Fin. ear. as.$\hat{2}$		-0.0619***	-0.0634***	-0.0635***	-0.0558***
Loans*Fin. ear. as.		0.0746***	0.0761***	0.0762***	0.0677***
Deposits$\hat{2}$		0.0328***	0.0329***	0.0329***	0.0320***
Capital$\hat{2}$		0.0814***	0.0840***	0.0840***	-0.0063
Bonds$\hat{2}$		0.0038***	0.0037***	0.0037***	0.0028***
Deposits*Loans		0.0557***	0.0549***	0.0549***	0.0316***
Deposits*Fin. ear. as.		0.0083*	0.0125**	0.0124**	0.0018
Capital*Loans		0.0054	0.0082	0.0082	0.0158*
Capital*Fin. ear. as.		-0.001	-0.0039	-0.0038	0.0059
Bonds*Loans		0.0082***	0.0080***	0.0080***	0.0058***
Bonds*Fin. ear. as.		-0.0038***	-0.0036***	-0.0036***	-0.0024***
Deposits*Capital		-0.1369***	-0.1399***	-0.1399***	-0.0829***
Deposits*Bonds		-0.0011	-0.0014*	-0.0014*	-0.0053***
Capital*Bonds		-0.0020**	-0.0018**	-0.0018**	0.0023**
Loans*Time			0.0008	0.0008	0.0007
Fin. ear. as.*Time			-0.0009	-0.0009	-0.0009
Deposits*Time			0.0053***	0.0053***	0.0060***
Capital*Time			-0.0038**	-0.0037**	-0.0037**
Bonds*Time			-0.0001***	-0.0001***	-0.0002***
Time				-0.0001	-0.0003
Time$\hat{2}$				-0.0002	-0.0001
Medium					-0.0589
Consumption					-0.2234***
State					0.1071***
Foreign					0.1436***
Public trading					0.0969***
Imacec					0.0004
M&A					0.0693*
L3.M&A					0.0394
L6.M&A					0.0126
L12.M&A					0.0066

*** Coefficients are statistically significant from zero at the 1% level. ** Coefficients are statistically significant from zero at the 5%. * Coefficients are statistically significant from zero at the 10%.

3 Efficiency at cluster level using SBM

As in SFA and DEA, overall inputs efficiency using SBM in Fig A1 sustains the tonic of Big banks at the top of the scores followed by Medium size and finally Consumption banks. There is a recent convergence in input efficiency between the most efficient groups while we obtain an increasing inefficiency on inputs at the Consumption cluster. Using cluster averages, the gap between Big and Consumption banks reached 70% in 2019.

Fig A1. Cluster average efficiency with SBM during 2000-2019.

4 Evolution of sight deposits by cluster

During 2000-2020, the share of sight deposits on total deposits doubled for Big banks, increased by almost 75% for Medium banks and more than ten fold for Consumption banks (Fig A2).

Fig A2. Sight deposits as a share of total deposits, cluster average.

5 Evolution of provisions and ROE by cluster

ROE in 2000-2019 broken down at cluster level, shows that in 2002 Consumption banks achieved market levels. Furthermore, after 2015, even with an important excess capital when compared to other clusters, this group persistently managed to locate at the top of the industry (Fig A3). This riskier profile is compensated with a provisions rate above that of system average (Fig A4).

Fig A3. Return on equity, cluster average.

Fig A4. Ratio provisions to assets, cluster average.

6 Efficiency ROE and market share, monthly data

In this section, we include our results for Granger causality tests using monthly data of market share (on assets) and the return on equity (dividends are paid once a year). The goal is to test whether efficiency gains are directed towards an increase in market share or to the accumulation of utilities. We used the Non Granger Causality test for up to 7 lags (maximum available according to sample). Note that all variables are stationary according to panel data Fisher-type test based on Augmented Dickey Fuller. Note that the lag time limit restricts us to test at most medium term relations, not long term plans.

Focusing on DEA results, summarized in Table A4, we find that efficiency dynamics Granger cause the return on equity but not otherwise. This means that efficiency gains or losses impact on results up to seven months afterwards. However, changes in the return on equity does not precede efficiency in the medium term. Maybe long term policies of efficiency gains take place in the long term but our test cannot capture them due to the size of the panel.

In addition, efficiency translates into market share changes although with a lag of three months. This means that pass through to prices, and then to market share, takes on average a quarter. On the other hand, it does not surprises us the observed causality from market share to efficiency as it is calculated as the portion of assets which are a component of efficiency estimate.

Table A4. Panel Granger Non-Causality test, $\tilde{Z}$ values. Monthly data.

10.a Test using DEA efficiency measure

**max lags** **x** **y**	**roe** **Eff. DEA**	**Eff. DEA** **roe**	**mktsh** **Eff. DEA**	**Eff. DEA** **mktsh**
1	1.5226	7.7342***	2.9686***	-0.174
2	1.1979	7.5348***	2.9604***	1.5097
3	1.5738	6.3546***	2.8197***	2.7788***
4	1.2482	5.9646***	1.8921**	1.4016
5	0.7784	4.4897***	1.0963	0.5343
6	0.3838	2.7089***	0.3265	0.1665

10.b Test using SFA efficiency measure

**max lags** **x** **y**	**roe** **Eff. SFA**	**Eff. SFA** **roe**	**mktsh** **Eff. SFA**	**Eff. SFA** **mktsh**
1	3.0542***	6.4882***	5.6045***	3.2891***
2	5.9765***	4.3632***	11.250***	-
3	4.3320***	4.6481***	6.3885***	9.3000***
4	10.650***	4.1248***	7.8388***	8.1300***
5	9.8847***	2.8708***	6.2827***	8.0473***
6	6.6788***	2.3482**	3.9061***	6.5972***

H_0 : x does not Granger cause y. H_1 : Granger Causality for at least one individual. $^{***}: p < 0.01, ^{**}: p < 0.05, ^{*}: p < 0.1$.

From a general perspective, results differ according the estimated efficiency series applied. In particular, SFA efficiency yields bidirectional causality relations in all variables while DEA efficiency renders unidirectional causality relations.
